# A new species of forest snake of the genus *Rhadinaea* from Tropical Montane Rainforest in the Sierra Madre del Sur of Oaxaca, Mexico (Squamata, Dipsadidae)

**DOI:** 10.3897/zookeys.813.29617

**Published:** 2019-01-07

**Authors:** Vicente Mata-Silva, Arturo Rocha, Aurelio Ramírez-Bautista, Christian Berriozabal-Islas, Larry David Wilson

**Affiliations:** 1 Department of Biological Sciences, The University of Texas at El Paso, Texas 79968-0500, USA The University of Texas El Paso United States of America; 2 Department of Biological Sciences, El Paso Community College, Texas 79968-0500, USA El Paso Community College El Paso United States of America; 3 Centro de Investigaciones Biológicas, Instituto de Ciencias Básicas e Ingeniería, Universidad Autónoma del Estado de Hidalgo, Carretera Pachuca-Tulancingo Km 4.5, Colonia Carboneras, C. P. 42184, Mineral de la Reforma, Hidalgo, Mexico Universidad Autónoma del Estado de Hidalgo Mineral de la Reforma Mexico; 4 Centro Zamorano de Biodiversidad, Escuela Agrícola Panamericana Zamorano, Departamento de Francisco Morazán, Honduras; 16010 SW 207th Avenue, Miami, Florida 33187-1056, USA Centro Zamorano de Biodiversidad Miami United States of America

**Keywords:** Morphology, new serpent species, Santa Catarina Juquila municipality, Southern Mexico, taxonomy, Morfología, nueva especie de serpiente, municipalidad de Santa Catarina Juquila, el sur de México, taxonomía

## Abstract

Content of the dipsadid genus *Rhadinaea* has changed considerably since Myers’ 1974 revision. Three species groups are recognized currently in the genus. Our fieldwork in Oaxaca in June 2018 produced a single specimen of *Rhadinaea* considered to represent a new taxon. This new species is described from converted Premontane Wet Forest in the municipality of Santa Catarina Juquila in the Sierra Madre del Sur of southern Oaxaca, Mexico. It is most closely related to *Rhadinaealaureata*, from which it can be distinguished easily by color pattern and scutellation, and represents a species group distinct from the other three occupying the genus.

## Abstract (es)

La conformación del género *Rhadinaea* ha cambiado considerablemente desde la revisión de Myers en 1974. Tres grupos de especies son reconocidos actualmente en el género. Nuestro trabajo de campo en Oaxaca en junio de 2018 produjo un espécimen de *Rhadinaea* que consideramos que representa un nuevo taxón. Describimos esta nueva especie de bosque húmedo premontano en el Municipio de Santa Catarina Juquila en la Sierra Madre del Sur en el sur de Oaxaca, México. Esta especie está más estrechamente relacionada con *Rhadinaealaureata*, de la cual se puede distinguir fácilmente por medio del patrón de color y escutelación, y representa un grupo distinto a los tres que forman parte del género.

## Introduction

The dipsadid genus *Rhadinaea* was once considered one of the largest genera of snakes in the Western Hemisphere. Myers’ (1974) well-known and well-regarded revision of this genus dealt with 45 species arranged into eight species groups. In the ensuing years, however, additional systematic work of a morphometric nature has reduced the content of *Rhadinaea*, largely by attrition, owing to allocation of a number of species placed in this genus by Myers (1974) to other genera. Myers (2011) resurrected the genus *Rhadinella* to contain the species he had placed earlier in the *Rhadinaeagodmani* group. The eleven species he included in this group in 1974 had been augmented to 15 by his 2011 paper and now comprises a total of 19 species, including 18 listed at the Reptile Database (http://reptile-database.reptarium.cz/; accessed 15 June 2018) and one other species, *Rhadinellastadelmani*, which was resurrected by Mendelson and Kizirian (1995), but not placed in the genus *Rhadinella* by Myers (2011), as noted by García-Vázquez et al. (2018). Savage and Crother (1989) placed the *Rhadinaealateristriga* group of Myers (1974), with seven species, along with the species of *Pliocercus* in the genus *Urotheca*. Other authors (Myers and Cadle 1994; Smith et al. 1995; McCranie 2011), however, rejected Savage and Crother’s conclusions in part, preferring to continue to recognize *Pliocercus* as a genus distinct from *Urotheca*. Finally, Myers and Cadle (1994) placed the *Rhadinaeabrevirostris* group of Myers (1974), with six species, in the resurrected genus *Taeniophallus*. As a result of the changes engendered in this systematic work over the last 44 years, *Rhadinaea* has been restructured to include only 21 species, arranged in five species groups (the *flavilata* group, containing *R.flavilata* and *R.laureata*; the *decorata* group, with *R.bogertorum*, *R.cuneata*, *R.decorata*, *R.forbesi*, *R.gaigeae*, *R.hesperia*, *R.macdougalli*, *R.marcellae*, *R.montana*, *R.myersi*, *R.nuchalis* [described by García-Vázquez et al. 2018], *R.omiltemana*, and *R.quinquelineata*; the *taeniata* group, with *R.fulvivittis* and *R.taeniata*; the *calligaster* group, with only *R.calligaster*; and the *vermiculaticeps* group, with *R.pulveriventris*, *R.sargenti*, and *R.vermiculaticeps*.

Two of the five species groups (*calligaster* and *vermiculaticeps* groups) currently recognized in *Rhadinaea* are extralimital to Mexico, occurring from Costa Rica to Colombia (Pérez-Higareda et al. 2002; Savage 2002; Köhler 2008; Ray 2017) and do not appear to contain taxa that are closely related to the one described herein. Another of the five groups (the *flavilata* group) comprises two species, one of which is distributed in the southeastern United States (*R.flavilata*) and the other in the Sierra Madre Occidental and the Trans-Mexican Volcanic Belt of western and central Mexico (*R.laureata*).

Apart from *R.laureata* mentioned in the previous paragraph, there are 15 other species distributed in Mexico (Heimes 2016; García-Vázquez et al. 2018), which are arranged in two species groups recognized by Myers (1974). These two groups are the *taeniata* group, with three species, and the *decorata* group, with 13 species. The distinctions among the three species groups of *Rhadinaea* represented in Mexico (*decorata*, *laureata*, and *taeniata* groups) are treated below in the Discussion section of this paper.

In general, species of the genus *Rhadinaea* are semifossorial, secretive, and infrequently encountered in the field, which means that significantly large sample sizes are difficult to impossible to accrue to assist in systematic studies and for other purposes. In June of 2018, however, while conducting general herpetofaunal surveys in the Sierra Madre del Sur of southern Oaxaca, Mexico, a single specimen of a *Rhadinaea* was encountered that we consider representing a species new to science, which we place in a new species group within this genus, and is described below.

## Materials and methods

We conducted fieldwork in the area around the type locality of the new species described herein in early June, 2018, at moderate elevations in disturbed premontane wet forest now used for the cultivation of coffee. The holotype was preserved in full-strength ethyl alcohol to allow for molecular study. It was deposited in the collection of the Centro de Investigaciones Biológicas at the Universidad Autónoma del Estado de Hidalgo, Mexico.

We examined and measured the holotype with a stereomicroscope and precision digital calipers to the nearest 0.1 mm. The format for the description generally follows that of Batista et al. (2016). The ventral scales were counted using the methodology of Dowling (1951). We used a slash mark (/) to delineate characters that differ from the left and right sides of the holotype. We used the following abbreviations for morphological measurements:

**SVL** (snout–vent length),

**TL** (tail length),

**TOL** (total length),

**HL** (head length),

**HW** (head width), and

**ED** (eye diameter).

The sex was determined by the presence of partially everted hemipenes. The color pattern in life was described based on the color catalogue of Köhler (2012).

## Systematic account

### Rhadinaea eduardoi sp. n.

Taxon classificationAnimaliaSquamataDipsadidae

http://zoobank.org/9D5AA496-27F7-4657-B1E9-59FA9901F81B

**Figure 1.**
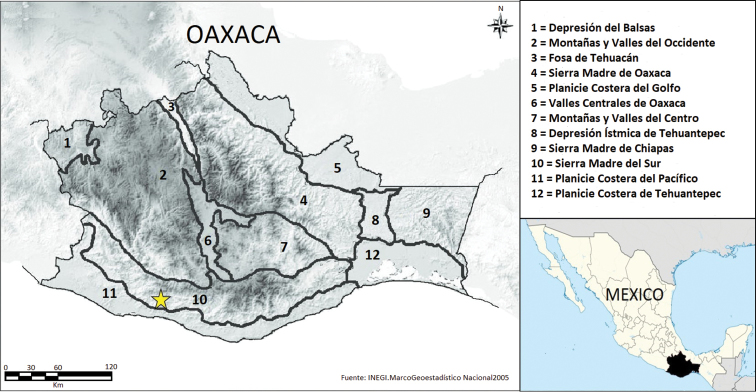
Map depicting the site (star) where *Rhadinaeaeduardoi* was found.

**Figure 2.**
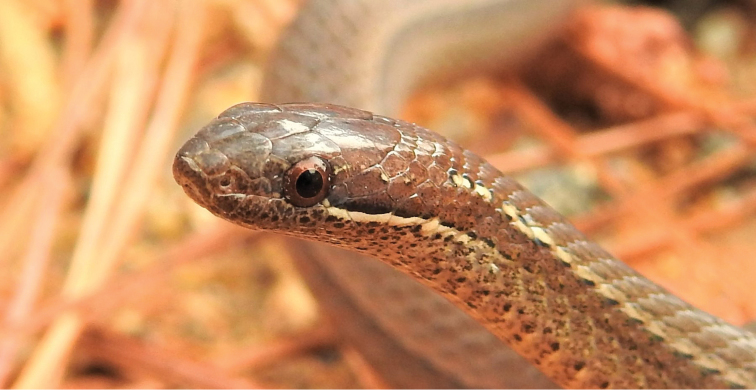
Head and anterior portion of body of holotype of *Rhadinaeaeduardoi*.

**Figure 3.**
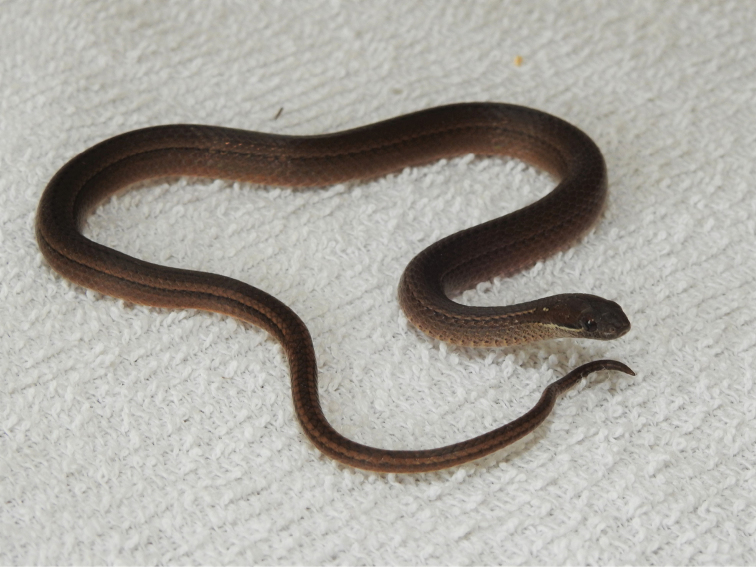
Holotype of *Rhadinaeaeduardoi* in life.

**Figure 4.**
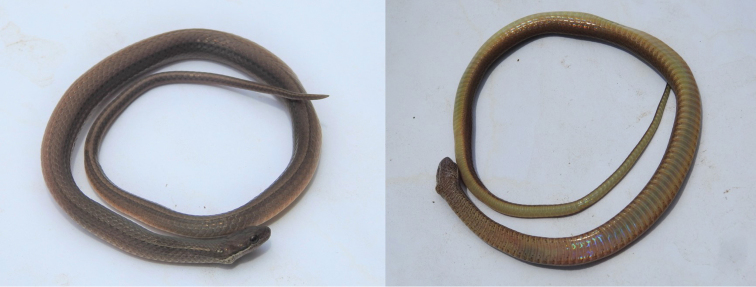
Doral and ventral views of the preserved holotype of *Rhadinaeaeduardoi*.

**Figure 5.**
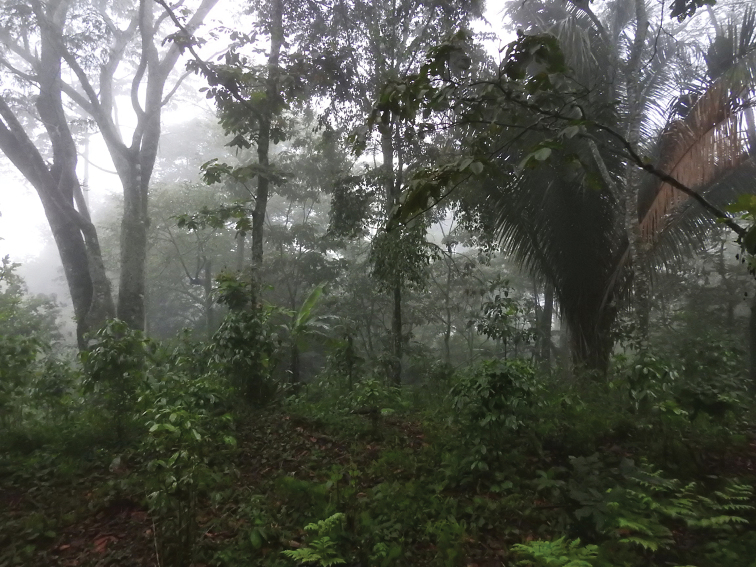
Habitat where holotype of *Rhadinaeaeduardoi* was found.

Figures 1 , 2 , 3 , 4 , 5 , Table 1

#### Common name.

English: Eduardo’s forest snake. Spanish: Hojarasquera de Eduardo

#### Holotype.

CIB-5457 (original field number VMS-2029), a subadult male from Mexico, Oaxaca, municipality of Santa Catarina Juquila, El Obispo, 1,320 m (UTM 681141.99, 1789988.05 [= 16.183573, -97.305614, datum WGS 84]), collected by Eduardo Mata-Silva on 6 June 2018 at 1800 hrs (Fig. 1).

**Table 1.** Distinguishing features of three species groups of *Rhadinaea* represented in Mexico (based on data in Myers 1974), compared to a new species group erected for the new species described herein.

*Rhadinaea* species groups in Mexico				
Distinguishing Features	*decorata*	*flavilata*	*taeniata*	*eduardoi*
Number of supralabials	Normally 8‒9, sometimes 7	Normally 7	8	7
Number of infralabials	10	9	10	8‒9
Subpreocular	Normally present	Absent	Usually present	Present
Head pattern	Conspicuous pale postocular marking extending from, or lying a short distance behind, upper rear edge of eye	Vague, sometimes scarcely discernible pale line outlined below by often diffuse, broken black line	Conspicuous pale stripe behind (and usually in front of) eye confluent with pale dorsolateral stripe	Conspicuous pale postocular marking extending from the lower rear edge of eye
Dark stripes	Variable, but with narrow dark line on rows 4 or 5	Diffuse or absent	Distinct, well-defined middorsal and lateral stripes	Diffuse, poorly defined middorsal and lateral stripes
Anal ridges in males	Usually present, but sometimes absent	Usually present	Usually present	Absent
Ventrals in males	110‒175	112‒167	140‒180	120
Ventrals in females	114‒186	118‒176	157‒197	‒
Subcaudals in males	56‒137	68‒97	83‒121	71
Subcaudals in females	60‒120	59‒92	78‒114	‒

#### Diagnosis.

A snake of the genus *Rhadinaea* that can be distinguished from all congeners by the following combination of morphological features: supralabials 7, with 3^rd^ and 4^th^ entering orbit; 120 ventrals; 71 subcaudals; one subpreocular (lower preocular); 17 dorsal scales throughout body; a head pattern lacking postorbital pale markings but having a pale line extending from the lower rear quadrant of the eye to the ultimate supralabial and slightly beyond, and a midbody dorsal color pattern of a lateral series of black dots in the lower apex of the scales of row V and a middorsal line confined to the middorsal scale row consisting of a series of disjunct spots on the posterior apex of otherwise dark brown scales.

#### Description of holotype

(Figs 2–4). Subadult male, as evidenced by size of the body and partially everted hemipenes; SVL 196 mm; TL 90 mm; TOL 286 mm, TL 31.5% of TOL; head slightly wider than body; HL 8.29 mm; HW 5.73 mm; ED 1.79 mm, about 21.6% of HL; rostral 1.86 mm long and 0.92 mm wide; internasal on right 1.15 mm long and 1.04 mm wide, on left 1.04 × 0.88, contacting anterior and posterior nasals, rostral, and one/both prefrontals; one large preocular subtended by a small subpreocular (lower preocular); two subequal postoculars; temporals 1 + 2, separating supralabials vi and vii from parietals; 7 supralabials, 1^st^ in contact with rostral, anterior and posterior nasals, and 2^nd^ supralabial, 2^nd^ in contact with posterior nasal (narrowly), 1^st^ supralabial, loreal, 3^rd^ supralabial, 3^rd^ in contact with loreal (narrowly), subpreocular, orbit, and 4^th^ supralabial, 4^th^ in contact with 3^rd^ supralabial, orbit, lower postocular, and 5^th^ supralabial, 5^th^ supralabial in contact with 4^th^ supralabial, lower postocular, and 6^th^ supralabial; 6^th^ supralabial in contact with 5^th^ supralabial, lower postocular (narrowly), anterior temporal, lower posterior temporal, and 7^th^ supralabial, 7^th^ supralabial in contact with 6^th^ supralabial, lower posterior temporal, and two posttemporal scales; 8/9 infralabials, 1^st^ pair in medial contact, four in contact with anterior chinshields, 5^th^ the largest; mental 1.29 mm long and 0.90 mm wide, separated from anterior chinshields by medial contact of 1^st^ pair of infralabials; anterior and posterior chinshields more or less subequal in size; four preventrals between posterior chinshields and 1^st^ ventral; smooth dorsal scales arranged in 17 longitudinal rows throughout the body, with no apical pits; 120 ventrals, cloacal scute divided; and 71 paired subcaudals between cloacal scute and terminal spine.

#### Coloration in life of holotype

(Figs 2, 3a, b). The dorsum and lateral portion of the head above the pale lip line is Cinnamon-Rufous (color 31). A white line begins posterior to the posteroventral quadrant of the eye and extends posteriorly to disappear on the neck. A white dash outlined above by black is present on the side of the neck about three scales posterior to the parietal scales and is separated from the pale line on the body. The region of the dorsum of the body above scale row five is Hazel (color 26). A disjunct black stripe is present on the middorsal scale row, consisting of a series of black dots, each of which markings occupies the posterior portion of each dorsal scale. The middorsal and lateral portions of the color pattern are separated by a narrow Pale Buff (color 1) line on scale row five that is bordered below by a black spot on the ventral apex of each scale. The lateral region of the dorsum is Cinnamon-Rufous (color 31) that becomes pale and flecked. The chin and anterior portion of the venter is dark gray flecked with black, grading to cream for the remainder of the venter. The lateral portion of each ventral on the anterior portion of the body bears a black spot.

#### Coloration in preservative

(Fig. 4). The dorsum of the head is dark brown. The lateral portion of the snout is very dark brown. The postocular and temporal region of the head is dark brown. The first four supralabials are dark gray, heavily mottled with very dark brown. The pale line on the upper portion of the postocular supralabials is white, bounded above by a black border and below by a broken black mottled border. A pair of longitudinally-arranged white spots bordered above by a black border are located just posterior to the temporal scales on the right side. These posttemporal spots are in line with a black-bordered white stripe and separated from this stripe’s point of origin by a single dorsal scale. The white supralabial stripe gradually disappears posterior to the head. The underside of the head is gray, heavily speckled with small black dots. This coloration continues onto the anterior portion of the venter, with the speckling decreasing in intensity. A black spot is present at the lateral apex of the ventral scales on the anterior venter. The dark speckling and spotting fades between ventrals, with the remainder of the venter and the ventral portion of the tail a pale yellow. The middorsal region of the body is dark brown. The middorsal row consists of a series of black dots positioned on the posterior apex of each middorsal scale. The dark brown scales of the middorsal and lateral regions of the body are separated by a pale line on the dorsal portion of scale row iv underlain by a black line on the ventral portion of the same scale row.

#### Etymology.

This species is named in honor of Eduardo Mata-Silva, collector of the holotype. Eduardo is the younger brother of the senior author of this paper, is a resident of Río Grande, Oaxaca, and is a highly valued member of our field crew working in Oaxaca. He also outshines the rest of the crew when it comes to finding snakes, as evidenced by his discovery of the holotype of the snake described herein.

#### Habitat and natural history observations.

*Rhadinaeaeduardoi* is resident in an area of converted Premontane Wet Forest, which presently supports a plantation of shade-grown coffee (Fig. 5). The holotype was found active at 1800 hrs on leaf litter approximately 10 m from a stream after a very light rain. Other herpetofaunal species encountered at this site were the anurans *Craugastorpygmaeus*, *Ptychohylaleonhardschultzei*, and *Exerodontasumichrasti*, and the lizards *Norops* sp. and *Holcosusundulatus*.

#### Distribution.

This species is known only from the type locality in the Sierra Madre del Sur of southern Oaxaca, Mexico (Fig. 1).

#### Conservation status.

*Rhadinaeaeduardoi* joins the extensive cadre of conservation category priority one species in Mexico designated by Johnson et al. (2017). This species is placed in this category due to its high EVS value and its restriction to a single physiographic region in Mexico. Its EVS can be calculated as 6 + 8 + 2 = 16, placing it in the middle of the high vulnerability category. This score is based on a contributory score of 6 for geographic distribution, because it is known only from the type locality, 8 for ecological distribution, because it is known only from a single forest formation, and 2 for human persecution, because it is semifossorial, non-venomous, and generally escapes human notice. It is restricted furthermore to the Sierra Madre del Sur. The EVS of 16 for this species matches that for *Rhadinaeabogertorum* (Johnson et al. 2017), another species of *Rhadinaea* endemic to the state of Oaxaca (Mata-Silva et al. 2015). Using reasoning similar to that employed by Batista et al. (2016), we opine that *Rhadinaeaeduardoi* can be placed in the Critically Endangered category, according to IUCN criteria B1ab(iii)+2ab(iii).

## Discussion

As noted in the introduction, Myers (1974) recognized eight species groups within the genus *Rhadinaea*, which has now been reduced to five, given that three of these groups have been allocated to other genera by subsequent authors (see introduction). Two of these five species groups, the *calligaster* and *vermaculaticeps* groups, are restricted in distribution to Lower Central America and have members remotely related to the members of the other three species groups, the center of distribution of which is in Mexico. The largest of these three groups is the *decorata* group, most species of which are restricted in distribution to Mexico, with one species (*R.decorata*) currently considered to range from Mexico to Colombia (Myers 1974). Another group of two species, the *laureata* group, contains one species found in Mexico and another distributed in the southeastern United States. The third group, the *taeniata* group, consists of three species, all restricted in distribution to Mexico. The principal distinctions among these three species groups are indicated in Table 1.

The new species described herein does not appear to belong to any of the three species groups of *Rhadinaea* represented in Mexico. It is distinguished easily from the members of the *decorata* group in having seven supralabials instead of eight or nine and eight or nine infralabials instead of 10. It differs from the members of the *taeniata* group in the same features (seven versus eight supralabials and eight or nine versus 10 infralabials), as well as having many fewer ventrals and subcaudals (120 and 71 versus 140–197 and 78–121, respectively). Although it agrees with the members of the *flavilata* group in having 7 supralabials, *R.eduardoi* has a subpreocular (lower preocular) and a strikingly different dorsal color pattern. In our opinion, *Rhadinaeaeduardoi* should be placed in a species group of its own (Table 1). Nonetheless, *R.eduardoi* appears to share its closest relationship with the members of the *flavilata* group, because of the presence of seven supralabials, with the 3^rd^ and 4^th^ entering the orbit. Logically, *R.laureata* would appear to be the most likely candidate as the closest known relative of *R.eduardoi*, but these two species are rather unlike one another in color pattern of the head and body, as well as in the much higher ventral numbers in male *R.laureata* (150–167, as opposed to 120 in the male holotype of *R.eduardoi*) and the much higher subcaudals in male *R.laureata* (86–97, as opposed to 71 in the male holotype of *R.eduardoi*). The color pattern of *R.laureata* consists of “a broad (three to five scale rows), gray dorsal stripe on a golden brown body, and little or no indication of lateral striping. There is a pale line through the top of the eye and another pale line that crosses the neck immediately behind the head; the last line may be confluent at its lower end with a line on the supralabials” (Myers 1974: 55). In addition, the distributional range of *R.laureata* is relatively remote from that of *R.eduardoi*, in the Trans-Mexican Volcanic Belt and the Sierra Madre Occidental, as opposed to the Sierra Madre del Sur.

Mexico is a country of significant herpetofaunal endemism, reported by Johnson et al. (2017) at 61.1% (on the basis of 789 endemic species of a total of 1,292 species). Mata-Silva et al. (2015) reported the state of Oaxaca to be the most speciose state in Mexico, with a total herpetofauna of 442 species. Of these 442 species, 164 are country endemics and 93 are state endemics. Combining these two figures indicates a total proportion of endemism in Oaxaca of 58.1% (257/442). The physiographic region in which *Rhadinaeaeduardoi* was discovered is the Sierra Madre del Sur (SMS), which Mata-Silva et al. (2015) reported as the second most speciose physiographic region in terms of herpetofauna of the 12 regions they recognized in the state. The number of species these authors reported in the SMS is 154, 34.8% of the 442 species known from the state at that time. This figure is second only to the number for the Sierra Madre de Oaxaca (216 species or 48.9% of the total of 442 species). The SMS regional herpetofauna was reported by Mata-Silva et al. (2015) to consist of 42 anurans, six salamanders, one caecilian, 46 lizards, 53 snakes, and six turtles. Of the 154 SMS species, 58 are country endemics and 25 are state endemics. The combined percentage of endemism for the SMS is 53.9% (83/154). Of the 53 species of snakes in the SMS, 18 are country endemics and three are state endemics; the combined percentage of snake endemism is 39.6% (21/53). The three state endemic SMS snake species are *Tantillaoaxacae*, *T.triseriata*, and *Micrurusephippifer*. Thus, *Rhadinaeaeduardoi* is an addition to this list, as well as to the overall list of endemic species in Mexico. *Rhadinaeaeduardoi* also joins two other species of *Rhadinaea*, *R.fulvivittis* and *R.myersi*, resident in the SMS of Oaxaca that are endemic to Mexico (Mata-Silva et al. 2015).

## Supplementary Material

XML Treatment for Rhadinaea eduardoi
